# Laundry Hygiene and Odor Control: State of the Science

**DOI:** 10.1128/AEM.03002-20

**Published:** 2021-06-25

**Authors:** Sarah E. Abney, M. Khalid Ijaz, Julie McKinney, Charles P. Gerba

**Affiliations:** a Department of Environmental Science, University of Arizona, Tucson, Arizona, USA; b Global Research & Development for Lysol and Dettol, Reckitt Benckiser LLC, Montvale, New Jersey, USA; c Department of Biology, Medgar Evers College of the City University of New York (CUNY), Brooklyn, New York, USA; Centers for Disease Control and Prevention

**Keywords:** laundry, hygiene, odor, pathogens, laundering

## Abstract

Laundering of textiles—clothing, linens, and cleaning cloths—functionally removes dirt and bodily fluids, which prevents the transmission of and reexposure to pathogens as well as providing odor control. Thus, proper laundering is key to controlling microbes that cause illness and produce odors. The practice of laundering varies from region to region and is influenced by culture and resources. This review aims to define laundering as a series of steps that influence the exposure of the person processing the laundry to pathogens, with respect to the removal and control of pathogens and odor-causing bacteria, while taking into consideration the types of textiles. Defining laundering in this manner will help better educate the consumer and highlight areas where more research is needed and how to maximize products and resources. The control of microorganisms during laundering involves mechanical (agitation and soaking), chemical (detergent and bleach), and physical (detergent and temperature) processes. Temperature plays the most important role in terms of pathogen control, requiring temperatures exceeding 40°C to 60°C for proper inactivation, while detergents play a role in reducing the microbial load of laundering through the release of microbes attached to fabrics and the inactivation of microbes sensitive to detergents (e.g., enveloped viruses). The use of additives (enzymes) and bleach (chlorine and activated oxygen) becomes essential in washes with temperatures below 20°C, especially for certain enteric viruses and bacteria. A structured approach is needed that identifies all the steps in the laundering process and attempts to identify each step relative to its importance to infection risk and odor production.

## INTRODUCTION

As highlighted in recent reviews, laundering plays an important role in the control of both pathogenic and odor-causing microorganisms (1, 2). Microflora will vary from one household, community, and region to another. Traditional laundering practices, socioeconomic factors, the availability of washing facilities, and the selection of products will influence many of these factors. Today, most laundry washing is conducted with machines (∼80%) (3), even in less-developed countries. However, handwashing is still practiced in developed countries, especially with delicate or non-machine-washable fabrics.

Most studies on laundering have focused their evaluation on practices within high-income countries, mostly involving machine washing. However, handwashing occurs in both high- and low-income countries to various degrees (3). Laundering procedures vary from region to region and are influenced by cultural practices and resources. Laundering involves a series of steps, independent of income status or machine access, each of which can affect the removal and diversity of dermally shed transient microflora within the textiles being processed. The goal of this review is to define laundering as a series of steps that influence the exposure of the person processing the laundry to pathogens and the removal and control of pathogens and odor-causing bacteria while taking into consideration the types of textiles. Defining laundering in this manner will help better educate the consumer and highlight areas where more research is needed and how to maximize products and resources.

## MICROFLORA OF CLOTHING

The microflora of laundry is important from the aspects of both preventing the transmission of diseases and odor control. The microflora of household laundry can be influenced by many factors, including textile attributions, handling, and usage (Table 1).

**TABLE 1:** Factors influencing the occurrence of bacteria/viruses/molds in laundry

Factor(s)	Description	Reference(s)
Fabric composition	Thickness, material, coloring agents; the thicker the fabric, the greater the survival of bacteria during laundering; greater survival of coliforms in hand/face towels after laundering and drying	S. K. Tamimi, S. L. Maxwell, L. Sifuentes, and C. P. Gerba, unpublished data; Gerba, unpublished
Storage conditions	Bacterial no. increases in hampers and if stored under high humidity (molds and total bacterial no.); we have found that clothing stored in hampers between laundering can result in the growth of bacteria in clothing	Kennedy and Gerba, unpublished
Usage	Location on body where worn (higher no. on undergarments and in pockets than on shirts; face and kitchen towels have higher no.); length of time worn; highest no. of enteric bacteria found in face towels and underwear (e.g., coliforms)	10; Gerba et al., unpublished
Season	Higher no. of bacteria during summer (mold); warmer weather and perspiration encourage growth of bacteria	K. A. Reynolds and C. P. Gerba, unpublished data
Age of clothing	Possibility of biofilm buildup; microorganisms adapt to repeated washing conditions and are not always removed	Reynolds Gerba, unpublished
Type of detergent	Additives to enhance detergent performance, i.e., enzymes and multiple surfactants	Reynolds and Gerba, unpublished
Dirt load	Type and quantity affect the performance of detergent and bleach	Kennedy and Gerba, unpublished
Wash temp and time	Greater survival of microbes at lower temp	45, 62; Kennedy and Gerba, unpublished
Drying temp and time	Greater survival at lower temp and shorter length of drying time	45; Kennedy and Gerba, unpublished
Air drying	Bacterial no. may increase in the clothing under humid outdoor conditions; prolonged exposure to sunlight may decrease no. of fungi	13
Type of microorganism	Resistance of microorganisms to washing varies with species and strain of microorganism; Mycobacterium, Enterobacter, and enteric viruses are more resistant to release from textiles and removal	13, 45; Kennedy and Gerba, unpublished
Concn of microorganisms in bodily excretions or secretions	Enteric viruses and bacteria can be excreted in high concn in feces; Salmonella occurrence at concn as high as 10^10^ bacteria/g and norovirus occurrence at concn as high as 10^11^ particles/g of feces	63
Concn of bodily excretions or secretions in clothing	The avg pair of adult underwear contains an avg of 0.1 g of feces	63
Method of washing	Machine washing versus handwashing; no data found on handwashing but expected to be less efficient	3
Quality of wash water	In developing countries, fecally contaminated water may be used, such as in streams	39

## MICROBES IN LAUNDRY

The source of most microbes in clothing is the human skin and bodily excretions and secretions. Activities such as cooking and eating, outdoor activities, and occupation can influence the distribution of the microbial flora present on the skin and within bodily excretions. Linens (bedsheets), cleaning tools (sponges, kitchen towels, and dishcloths), and bath towels can have their unique microflora. Each group of textiles has unique features such as types of fabric, usage, and dirt load. This also influences the occurrence of pathogens and odor-producing bacteria within laundry.

### Pathogens.

Epidemiological studies have suggested the role of fabrics in transmitting infectious agents in facilities (4). Since most pathogens associated with textiles have multiple transmission routes, tracing epidemiological associations with laundering and transmission is difficult. One study suggested the spread of respiratory illness associated with public laundromat usage and not using bleach during laundering (5).

Numerous pathogens have been detected in textiles before laundering (Table 2). Any pathogen associated with human illness is likely to be found in clothing and most other textiles. Outbreaks of illness have been associated with textiles contaminated with pathogens (viruses, bacteria, and fungi, where most cases are associated with health care workers and facilities [1, 6, 7]).

**TABLE 2:** Some pathogens detected in textiles before washing

Organisms^*a*^	Reference(s)
Bacteria	
Salmonella enterica serovar Typhimurium	1
S. enterica serovar Hadar	1
Acinetobacter baumannii	1
MRSA	1
Bacillus cereus	64
Clostridium difficile	33, 42
Neisseria gonorrhoeae	1
Vancomycin-resistant enterococci	42

Fungi	
Microsporum canis	1
Sarcoptes scabiei	64
Alternaria alternata	65
Trichophyton mentagrophytes	13

Viruses	
Hepatitis B virus	1
Hepatitis A virus	66
Papillomavirus	67
Rhinovirus	68
Adenovirus	69
Influenza virus	68
SARS-CoV-2	58
Parainfluenza virus	68
RSV	68
Rotavirus	70

Helminths and protozoa	
Pinworms	18

a RSV, respiratory syncytial virus.

### Bacteria.

Pathogenic bacteria causing enteric disease (Salmonella) and skin infections (MRSA [methicillin resistant Staphylococcus aureus]) have been associated with textiles. In one study, Salmonella was detected in 15% of household sponges in the United States and 3% of hand/face towels (8). Escherichia coli and other enteric bacteria were also common. E. coli has also been detected in reusable grocery bags (9). Fecal bacteria are common in undergarments of both children and adults (10). Under conditions of storage (hamper or closet) before or after laundering, bacterial numbers can increase (11).

### Fungi.

It has been suggested that fungi present in clothing may also play a role in the transmission of dermatitis and onychomycosis (infection of the nails) (12). Fungal pathogens have been isolated from patients suffering from tinea pedis (13). Clothing has been linked to the transmission of Microsporum canis (1). M. canis belongs to the group of dermatophyte fungi, which are closely related microorganisms that can invade the stratum corneum of the epidermis and keratinized tissues derived from it, such as the skin, nails, and hair of humans and animals. These fungi produce an infection called dermatophytosis, commonly referred to as ringworm or tinea. Of 70 household washing machines sampled in one study, 79% were positive for fungi (14). Among the species detected, the opportunistic fungi *Candida* and Fusarium were detected. Fungi can also cause life-threatening infections among the immunocompromised. Mucormycosis, an infection of the order *Mucorales*, can cause mortality rates that can exceed 50% (15). Outbreaks have been associated with linens in health care (16). It was found that 33% to 73% of recently laundered linens were contaminated with Aspergillus (16).

### Protozoa.

While no studies on the occurrence of protozoa in fabrics could be found, they can be expected to be present in fecally soiled clothing from infected individuals as well as individuals who work with animals, such as farmers, cattle operators, and veterinarians. In an outbreak of cryptosporidiosis, a wife was infected through washing her husband’s soiled veterinary clothing (17).

### Viruses.

A wide range of enteric and respiratory viruses have been detected in textiles, including rotavirus, hepatitis A and B viruses, herpes simplex virus, severe acute respiratory syndrome coronavirus 2 (SARS-CoV-2), influenza virus, HIV, and papillomavirus (Table 2). A blood-borne pathogen, hepatitis B virus has been transmitted by sharing bathroom towels. Hepatitis A virus and vaccinia virus (smallpox virus) have also been shown to be transmitted by textiles (1).

### Helminths.

Fecally contaminated clothing and fabrics can also be expected to contain helminths (worms) and their eggs. These include tapeworms, *Ascaris*, and pinworms, etc. Good hygiene and water and food supplies have resulted in a low incidence of most intestinal worm infestations in the developed world. Pinworms are the most common helminth in the United States and Western Europe, with prevalence rates in some communities being as high as 30% to 50% (18). It has been suggested that pinworm (Enterobius vermicularis) contamination of bed linen and clothing could be involved in their transmission (18). Pinworm and *Ascaris* eggs can survive for 2 to 3 weeks on clothing and bed linens (19, 20). No data on the occurrence of helminths in clothing and fabrics could be located.

## SURVIVAL OF PATHOGENS IN LAUNDRY

The survival of microorganisms in stored-before-laundering fabrics depends on several factors, including relative humidity (RH), temperature, and material. Proceeded by a lower rate of inactivation, most microbial inactivation takes place during drying of the original suspension that contains the microorganism (e.g., saliva or mucus). Drying of respiratory viruses results in a usual 10- to 100-fold reduction in titers (21). Some microorganisms survive better at certain relative humidities than others. For example, rotavirus survives well at high (85% ± 5%) and low (25% ± 5%) relative humidities on cotton-polyester (22). The type of material and the presence of dyes or coloring agents may also affect the persistence of microorganisms on/in textiles (23, 24; C. P. Gerba, unpublished data). Water loss was observed to be greater in more hydrophobic fabrics. Certain fabrics such as cotton towels hold moisture to a higher degree, reducing drying and allowing the potential growth of bacteria and mold (C. P. Gerba and L. Sifuentes, unpublished data). Kampf (21) found that bacteria at room temperature survived the longest on polyester (up to 206 days), compared to 90 days in cotton and mixed fibers. Most bacteria were found to survive better at higher relative humidities. Enveloped viruses survived for less than 1 day on cotton fabrics, while they survived for 7 to 12 days on polyester. The thickness of the clothing/fabric may also affect drying and cause the regrowth of bacteria, such as coliforms in face towels (Gerba, unpublished). Dyes used in the manufacturing of fabrics may also have antibacterial activity (23).

Most respiratory viruses, including SARS-CoV-2, do not survive more than a day or two in clothing (25, 26). The survival of influenza virus in clothing was found to be related to the rate of water loss during drying (24). The thickness of the cloth and its color were related to survival, with faster inactivation in black cloth. However, some enteric viruses, such as rotavirus and hepatitis A virus, may survive for weeks (25, 27).

Pathogenic bacteria and molds, such as Salmonella and MRSA, may survive for weeks in clothing (1). Naturally occurring Pseudomonas aeruginosa and Acinetobacter spp. can grow in clothing even after laundering the clothing of wastewater treatment workers (28). Apparently, naturally occurring bacteria have adapted to laundering conditions, and enough organic matter remains for their regrowth during storage after laundering.

At a temperature of 25°C and an RH of 50%, 21 days were required for a 90% reduction in Giardia cysts in soiled 100% cotton (29). Under the same conditions, *Cryptosporidium* oocysts required ∼60 days. Furthermore, Entamoeba histolytica cysts, pinworm (*Enterobius vermicularis*), and Ascaris suum ova at temperatures of <25°C and 50% RH required 21, 26, and 188 days for 3-log_10_ reductions in soiled 100% cotton (30).

## REMOVAL OF PATHOGENS AND ODOR-CAUSING MICROBES BY LAUNDERING

**FIG 1:**
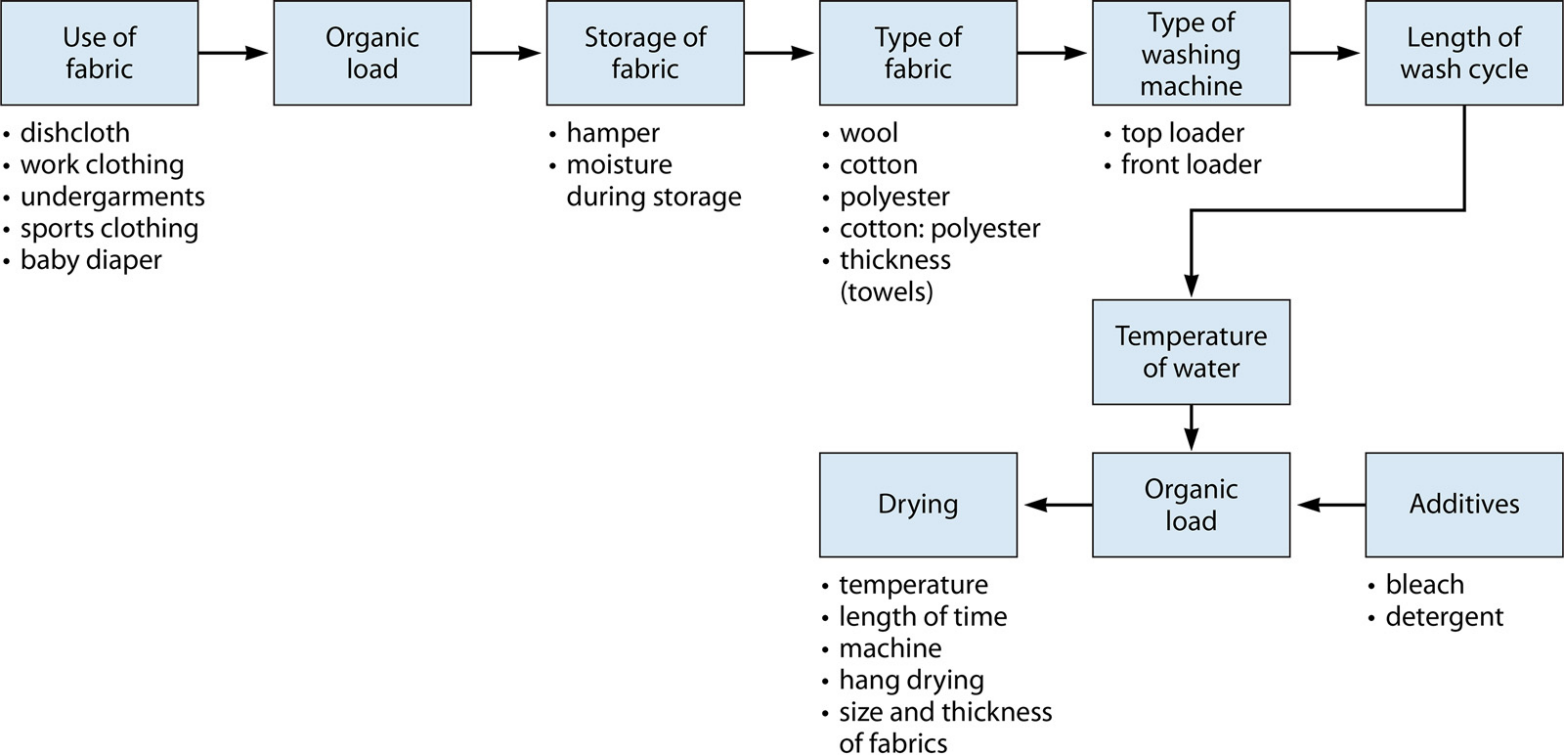
Factors influencing the removal of microorganisms by laundering.

The removal of microbes by the laundering process depends upon several factors, as illustrated in Fig. 1. As can be seen, many factors may influence the removal (detachment and/or inactivation) of microorganisms but also the potential for contamination (e.g., occupation, such as a wastewater worker versus a schoolteacher). It is likely that these factors also result in the establishment of a resident microflora adapted to combinations of these factors. This is important to note as processes intended to detach and inactivate microorganisms may introduce additional microbial communities (e.g., resident microflora from washers and dryers) and may influence malodors.

The removal of pathogens from the laundry is largely dependent on washing and drying practices. The reduction-release and/or inactivation of pathogens is influenced by detergent selection, other additives (bleach), water temperature, and drying. In North America, cold-water washes, using water from a cold-water tap, are commonly practiced. Enveloped viruses such as SARS-CoV-2 and influenza virus are very sensitive to the removal/inactivation capability of some detergents, which can result in the elimination of these viruses even in cold-water washes (31, 32). The median cold-water wash temperature is 14.4°C (57.9°F) in the United States. It has been recommended that temperatures of 40°C to 60°C (104°F to 140°F) and/or the use of bleach is needed for more resistant enteric and dermal pathogens (1, 33). Drying also provides an additional barrier to transmission/survival, with both the temperature and duration playing a role in disinfection (C. P. Gerba and D. Kennedy, unpublished data). Higher-temperature settings and longer drying can significantly reduce microbial numbers. Acinetobacter baumannii and Staphylococcus aureus are among the non-spore-forming bacteria that are the most resistant to drying and heat found in the laundry. Washing at temperatures above 60°C is necessary to achieve a 99.9% reduction (35). Mycobacterium, fungi, and enteric viruses (hepatitis A virus, adenovirus, and rotavirus) also require higher washing and drying temperatures for significant reductions (1, 36; Gerba and Kennedy, unpublished). The use of activated-oxygen bleach (AOB) in cold-water washes (<40°C) will significantly reduce the levels of bacteria and viruses but may not eliminate them (2, 35). Gerba and Kennedy (unpublished data) found that AOB was more effective in reducing Mycobacterium and enteric viruses than chlorine bleach in the presence of detergent, probably because both the high pH of detergent and the presence of dirt loads adversely impacted chlorine bleach activity.

## STEPS IN LAUNDERING

Machine laundering is a series of steps involving sorting of cloths, loading of the washer, removal from the washer, drying, and storage. Handwashing of textiles may involve washing in a basin, public facility, or surface water source (river or reservoir). Each step in the process results in the exposure of the individual handling the clothing to pathogens in the clothing and potentially to any present in the water. Exposure may occur by both contamination of the hands and microbial aerosols (37). Today, machine laundering is the most common method of practice, but hand laundering is practiced in all regions of the world (3). In high-income countries, this is usually limited to fine fabrics or other items for which machine washing is not recommended (e.g., reusable grocery bags made of plastic fibers). In North America, 82% of the laundry is machine washed, while in Africa and the Middle East, only 45% is machined washed (3). From 6% to 14% of household laundry is still handwashed depending upon the region of the world.

## HANDWASHING OF LAUNDRY

Delicate fabrics, such as lingerie and reusable grocery bags, can be significantly contaminated with bacterial and viral pathogens (9, 38; Gerba, unpublished). The use of sinks or plastic basins, often used for handwashing of fabrics, can result in contamination of the hands and cross-contamination if multiple items are washed. In addition, such items are not washed at temperatures as high as those in washing machines and are hang dried, not reaching temperatures reached in machine dryers.

**FIG 2:**
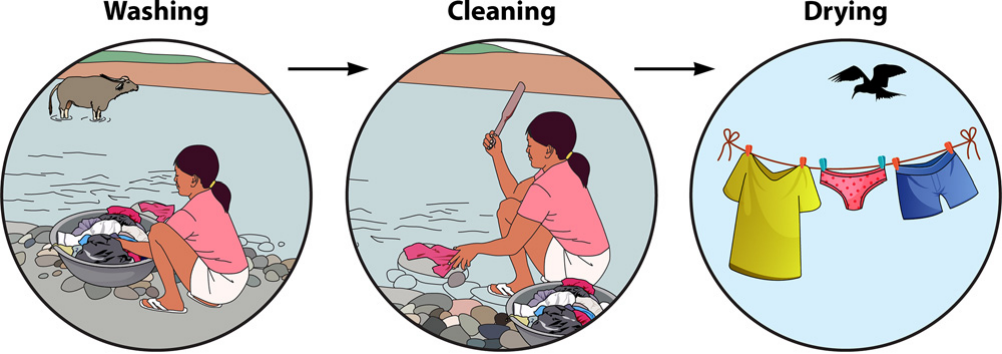
Steps in handwashing as practiced in some regions of the developing world.

In low-income countries, laundry bar soap is often used for washing instead of detergents, which may reduce the efficacy of the processes in terms of dirt removal and microbes (Fig. 2). In addition, water with fecal contamination may be used. A study in India suggested that handwashing of laundry in fecally contaminated rivers was a potential risk factor for the transmission of hepatitis E virus (39).

## LAUNDRY PROCESSING

**FIG 3:**
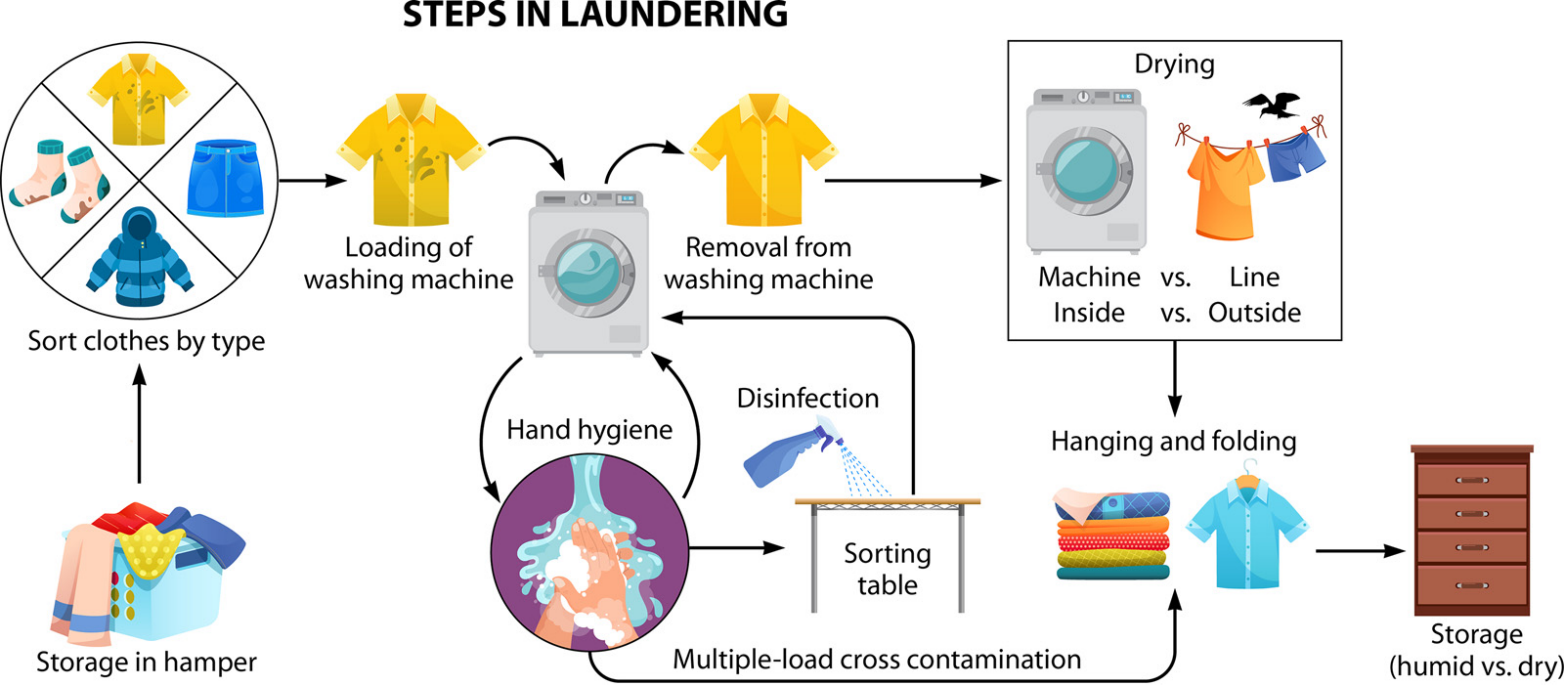
Steps involved in home laundering using a washing machine.

Laundry processing involves several steps, as shown in Fig. 3. Each step not only involves potential exposure to pathogens but also can affect the overall microbial load, including odor-producing bacteria.

Storage of the laundry in a hamper or humid environment can result in the growth of odor-producing bacteria, molds, and, potentially, pathogenic bacteria (11, 40). The soil load may also enhance the potential for the growth of these microorganisms. Bacteria and fungi may survive for weeks to months in textiles (11). Enteric bacteria and viruses are also capable of prolonged survival (weeks), with some respiratory viruses surviving for at least several days (1, 25).

Sorting of the textiles could result in both cross-contamination of the textiles, the surfaces where it is conducted, as well as the hands of the person doing the sorting and aerosolization of pathogen-associated particulates. The same is true for loading of the washing machine, removal of the laundry from the washing machine, and placement in the dryer or hanging of the clothes to air dry (Gerba, unpublished). The dry heat from the dryer can result in a significant reduction of microbes. Drying in the outdoor environment may result in a reduction of microorganisms from the UV rays in sunlight. Still, the presence of humid air conditions could result in the regrowth of some microorganisms, including recontamination events from bird droppings. Sorting prior to hanging of the clothes can result in additional exposure. Sorting of the clean clothes in the same area as the one used for sorting of the unwashed clothes can result in additional cross-contamination. Also, handling dirty clothes and then sorting washed clothes can result in cross-contamination. This cross-contamination web has been reported in commercial laundries (41, 42). Furthermore, storage of the clothing can result in the growth of bacteria and molds under humid conditions (28).

### Pathogens.

The removal of pathogens from the laundry is largely dependent on washing (release and/or inactivation) and drying (inactivation) practices. The reduction of pathogens is influenced by detergent selection, other additives (bleach), water temperature, and drying (43). In North America, cold-water washes are the common practice, while in Europe, hot-water washes are much more common; hot-water taps in the United States alone are recommended to be set at a maximum of 49°C (120°F) to 52°C (125°F) to avoid scalding (44). In Europe, the temperature on the washing machine is selected by the user, and hot-water washes of >40°C are the usual practice (1). It has been recommended that a minimum wash temperature of >40°C is necessary to impact the detachment and sterilization of pathogens from laundry where AOB detergents are not used (1). Wash temperatures of 60°C are recommended for fungi (13). Microorganisms with greater resistance to removal by washing and drying include spore-forming bacteria (Clostridium difficile), Mycobacterium spp., Bacillus cereus, Acinetobacter spp., Aspergillus and other fungi, hepatitis A virus, adenovirus, rotavirus, and enteroviruses (45; C. P. Gerba, S. Maxwell, L. Y. Sifuentes, and A. H. Tamimi, submitted for publication). Table 3 illustrates the removal of different types of microorganisms by machine washing.

**TABLE 3:** Log_10_ reductions by machine drying temperature and duration

Organism(s)	Log_10_ reduction	Method, drying temp, and time	Reference
Rotavirus	0.32	Permanent cycle, 55°C, 28 min, cotton sheets	45
Hepatitis A virus	0.29		
Adenovirus	1.36		
S. aureus	2.89–2.50	Huebsch gas dryer, medium temp, 16 min, cotton-polyester sheets	71
S. aureus	3.23	Cotton-polyester, 10 min, 46°C, 20 min	72
Serratia marcescens	>3.84		
Bacillus stearothermophilus	0.73		
S. aureus	1.82	Permanent press cycle, 55°C, 28 min, cotton	Gerba and Kennedy, unpublished
E. coli	>4.16		
*S.* Typhimurium	4.83		
Mycobacterium fortuitum	0.14		

Naturally occurring bacteria	0.5–1.0	175.6°C–177.8°C, 2 min	73

Enveloped viruses such as SARS-CoV-2 and influenza virus are very sensitive to the inactivation action of detergents, which can result in the elimination of these viruses even in cold-water washes. However, viruses and some bacteria and fungi may require hot-water washes, bleach, and high settings on dryers (31, 45). Heinzel et al. (31) found that while enveloped viruses were inactivated by >99.99% as a result of washing textiles at 20°C, temperatures of 30°C to 40°C in addition to a sanitizing detergent (AOB) were necessary for the inactivation of nonenveloped viruses. Both chlorine bleaches and activated-oxygen sanitizers result in increased reductions of pathogens in textiles (1, 35). Activated-oxygen bleaches are common in detergents used in Europe but not the United States (1). While bleach effectively reduces the number of bacteria and viruses, AOB was found to be more effective in simulated washing loads (2, 6, 46). This may be because of the high pH caused by the laundry detergent resulting in a lower efficacy of the bleach.

Recently, Zinn and Bockmuhl (47) found that the addition of acetic acid (final concentration, 0.75%) to a wash load of soiled fabrics and detergent reduced Pseudomonas aeruginosa, E. coli, and Staphylococcus hominis by more than 7 log_10_ units, while S. aureus was reduced by 5.8 log_10_ units. Such approaches may be useful in reducing pathogens in resource-limited regions of the world as well as high-income regions.

### Odor-producing microorganisms.

Bacteria and fungi are the major causes of malodors in clothing. Odor-producing bacteria and fungi may originate not only from use but also during storage or from cross-contamination between articles, the washing machine, and even machine or hang drying (40, 48, 49). During slow air drying, these microorganisms may increase in numbers (48). Greater malodors are more often associated with polyesters because the odor-producing hydrophobic compounds attach more strongly to these fabrics than to cotton and are more difficult to remove by detergents alone (48).

Washing machines develop a unique biofilm influenced by detergents and high temperatures. Thermophilic bacteria are more common in washing machines and clothing as a result (50, 51). Proteobacteria are the predominant phylum of bacteria in washing machines (51). Pseudomonas putida was found to be the most resilient biofilm former in washing machines (52). Washing machines are believed to be a significant source of bacteria and fungi that cause malodors in laundry (49). Kubota et al. (40) reported that the species Mycobacterium osloensis was primarily responsible for malodor in laundry. They found that it had the potential to generate the odor compound 4M3H (4-methyl-3-hexenoic acid) as well as a high tolerance to desiccation and UV light.

Overall, it appears that the major causes of malodors are bacteria and fungi that can survive laundering. Upon wetting, these bacteria can grow both in the washing machine itself and within textiles.

## IMPACT OF WASHING MACHINES ON MICROFLORA OF TEXTILES

### Type of textiles.

The ability to release microbes from textiles by washing is influenced by their structure, fabric type, and thickness. For example, bath and face towels make it more difficult to remove bacteria because of their thickness. Coliform bacteria within bath towels have been found to survive washing in hot water and extended drying (Gerba, unpublished). This suggests that the occurrence of odor-causing bacteria may be greater in some types of materials than others (i.e., sponges and bath and kitchen cleaning towels) (53).

### Type of washing machine.

Front-loader machines have become more common since they reduce water usage and are more efficient. However, residual water that remains in the machine may affect odors and result in cross-contamination of laundry. In a survey of washing machines in homes, ∼20% were found to harbor E. coli in the drum (Gerba, unpublished). Fungal pathogens such as *Candida* and Fusarium species have been detected in residential washing machines (14).

### Exposure.

The greatest exposure to pathogens occurs from handling the soiled laundry before it is placed in the washing machine and handling the washed laundry when either putting it in the dryer or hanging it to dry. The contamination of the hands during these events can lead to infection of the individual handling the laundry. This is especially true for enteric pathogens since movement of the hands to the mouth (lips) results in direct access to the intestinal tract. Contact with contaminated skin can result in the transmission of skin infections, and respiratory infections can be transmitted from contact to the nose, eyes, and mouth.

**FIG 4:**
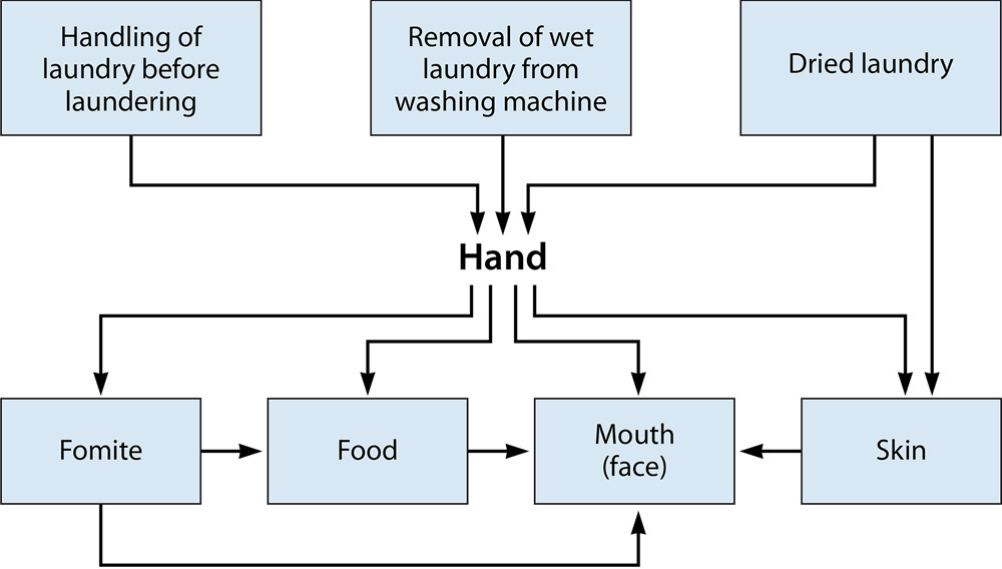
Exposure events for handing and washing laundry.

The next greatest exposure results from removing the laundry from a dryer or during collection after it has been hung to dry. Finally, the reuse of the fabric results in a potential additional exposure. Exposure events from handling laundry are shown in Fig. 4. The microorganisms may be transferred from the hand to the face, other fomites, and food. We consider that the greatest risk is likely from hand-to-mouth contact from directly handling the laundry.

Only a certain percentage of the microorganisms on the fabric is transferred to the hand from contact with each other (54). The number transferred to the hands may depend upon the type of fabric, the moisture content of the fabric, and the gripping strength of the individual. Exposure will also depend upon how many contaminated fabrics are handled and how many times the face or mouth is touched (55). Generally, less transfer of virus occurs from fabrics than from hard nonporous surfaces. Lopez et al. (56) found that 0.03% of MS2 virus was transferred to the hands from dry cotton fabric at low relative humidity (15 to 32%) and that 0.3% was transferred at high relative humidity (40 to 65%). Indoor humidity in the United States ranges typically from 40% to 60%. In contrast, the rate of transfer of MS2 from hard surfaces (stainless steel) to the finger was 21% to 79% depending upon the relative humidity (56). Rusin et al. (54) found that only 0.005% of the bacterial virus PRD1 was transferred from dry cotton cloth to the hands. Alternatively, the rate of transfer was 0.0005% from a cotton-polyester fabric. From a moist wet cotton dishcloth, it was 0.03%. No data on the transfer of rotavirus to hands on fabrics could be found. The rate of transfer of human rotavirus from a stainless steel surface to the finger was found to be 16.6% (57). Rusin et al. (54) found that 33.9% of the coliphage PRD1 virus was transferred from the hand to the mouth. No data could be found on the transfer of enveloped viruses from fabrics to hands or from hands to face. Such information would help better define risks from handling laundry from persons with respiratory infections, including SARS-CoV-2-associated coronavirus disease 2019 (COVID-19). Recently, SARS-CoV-2 RNA was detected from a sheet, a duvet cover, and a pillow cover, further highlighting the paramount importance of proper handling procedures during the replacement and/or laundering of used clothing of SARS-CoV-2 patients (58).

## CONCLUSIONS AND RESEARCH NEEDS

The goal of laundering is to control both the exposure to pathogenic microorganisms and odor. Both are interrelated, and the control of one implies the control of the other. One of the biggest problems in assessing the efficacy of these goals for domestic laundering processes is the lack of a consistent, structured approach to all the factors involved. A structured approach is needed that identifies all the steps in the process and attempts to quantify both risks of infection and mitigation of odor. One factor that needs to be assessed is the strategies in the home that can be used to minimize environmental impacts (energy usage) while still minimize odor and exposure to illness-causing microbes. The use of low temperatures during laundering may require additional strategies such as the use of a sanitizer and/or extended machine drying, especially when certain enteric viruses and bacteria may be present. There is also a need to consider the laundering of work and professional clothing, demographics of the household, regional differences in laundering practices, and types of textiles. All of these are needed to provide guidance to households to maximize the benefits of laundering.

**FIG 5:**
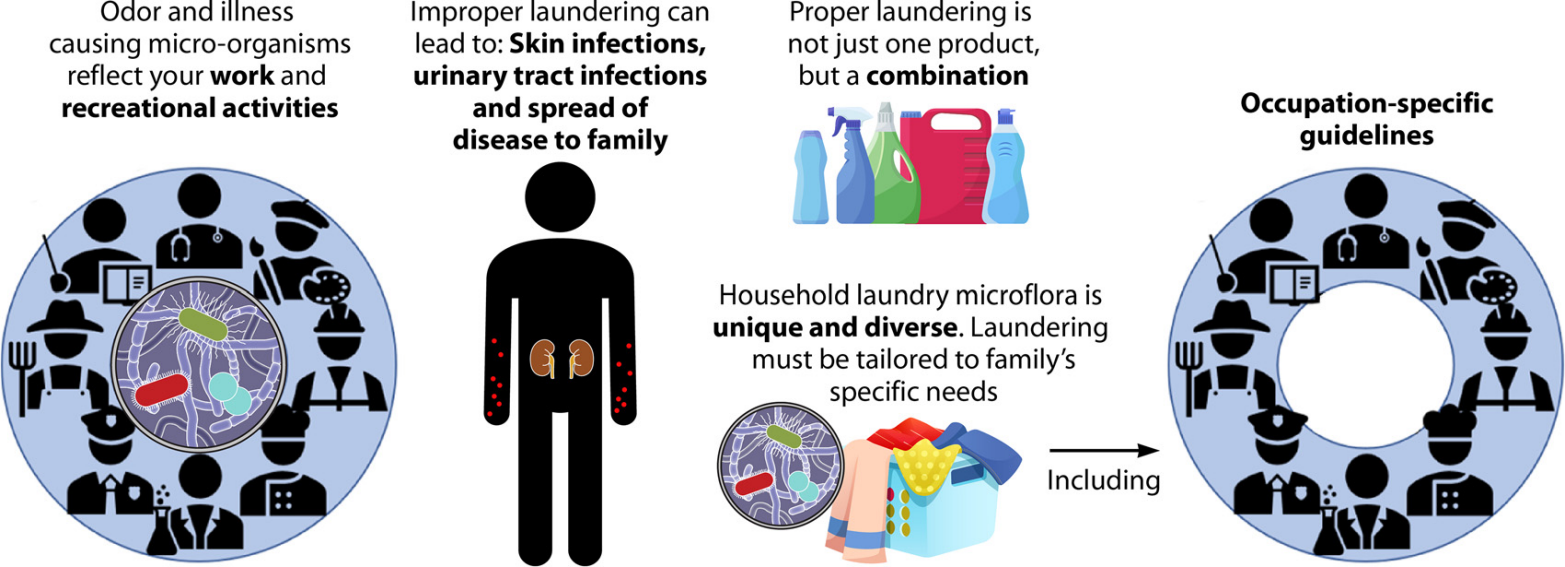
Research studies to better define and communicate risks associated with laundering.

Research should be focused on providing information that can be used to identify risks and how they can be reduced in a more quantitative fashion. Using event trees to define the impacts of each process in laundering and quantitative microbial risk assessment (10, 59) can quantify the impact of each process in terms of odor reduction and risk of infection. These can then be used to develop guidance for domestic laundering, which is not yet available and would have the greatest benefit. In fact, there are no understandings of a definition for what it means to achieve a “safe” level of risk reduction in laundering practices. Research on the efficacy of machine washing alone has only recently been detailed (60, 61). Figure 5 illustrates these needs. More information on the types and concentrations of odor-causing and pathogenic bacteria in the laundry can be used to better define strategies for processing while also taking into consideration the demographics of the household with respect to the types and coarseness of textiles (professional clothing, thickness, and use). In this regard, the best combination of products can be selected. Better information on the impact of storage before and after laundering of textiles is also needed. We are now in an age of increasing concern about the spread of emerging pathogens and means of prevention and control, particularly with the ongoing SARS-CoV-2-associated COVID-19 pandemic. With additional information through future research endeavors, we can provide the best laundering options to ensure a healthy household.
